# Parametric study and fuel quality assessment of biofuel from hydrothermal liquefaction of microalgae grown in municipal wastewater

**DOI:** 10.1038/s41598-025-33818-6

**Published:** 2026-01-14

**Authors:** S. H. Hassan, N. K. Attia, G. I. El Diwani, Sh. K. Amin, Sayeda M. Abdo, Fatma H. Ashour, Ehab F. Abadir

**Affiliations:** 1 Chemical Engineering Department, Faculty of Engineering, Cairo University, Giza, Egypt; 2 Chemical Engineering and Pilot Plant Department, National Research Centre (NRC), Dokki, Giza Egypt; 3 Water Pollution Research Department, National Research Centre (NRC), Dokki, Giza Egypt

**Keywords:** Hydrothermal liquefaction, Microalgae grown in municipal wastewater, Biofuel yield, Reaction study, Renewable energy, Fuel quality., Engineering, Chemical engineering

## Abstract

Hydrothermal liquefaction (HTL) of algal biomass is a promising approach for renewable biofuel production. The actual study investigates the effects of reaction temperature (225–325 °C), residence time (15–60 min), algae-to-water mass ratio (1:5–1:20), and pressure on the yield and quality of biofuel derived from municipal wastewater-grown mixed algal-cyanobacterial biomass. Eleven HTL experiments were conducted, and the resulting products were separated into gas, liquid, and solid phases for thermal and chemical analyses. Selected biofuel samples were characterized using gas chromatography–mass spectrometry (GC–MS), elemental analysis, and thermogravimetric analysis (TGA). The biofuels contained complex mixtures of aliphatic hydrocarbons, aromatics, phenolics, carboxylic acids, esters, and nitrogen-containing compounds, classified into biogasoline, bio-jet fuel, biodiesel, and motor oil fractions. Optimal yields of biofuel, gas, and solid residues were 16.86%, 26.14%, and 40.43%, respectively, achieved at a 1:10 algae-to-water ratio, 30 min reaction time, and 250 °C. The biofuel composition comprised 11.37% gasoline, 29.41% kerosene, 9.71% diesel, with a heating value of 42.93 MJ·kg⁻¹. A higher fraction of gasoline, kerosene and diesel-range compounds enhances energy density and combustion stability, while lower oxygen and nitrogen content improves storage and fuel properties. Solid residues exhibited uniform physical properties but were unsuitable for high-grade biochar due to low carbon and high inorganic content. These findings demonstrate that HTL of municipal wastewater-grown microalgae is a viable route for sustainable biofuel production, integrating resource recovery with renewable energy generation, while systematically evaluating key operational parameters and characterizing the resulting biofuel for downstream applications.

## Introduction

The rising global energy demand and the environmental impacts of fossil fuel consumption have intensified the need for sustainable alternatives^1^. Global CO₂ emissions from fossil fuel combustion have increased from 6 Gt yr⁻¹ in 1950 to nearly 37 Gt yr⁻¹ in 2023–2024, elevating atmospheric CO₂ from ~ 280 ppm to over 420 ppm and contributing to global warming already exceeding 1.5 °C^2,3^. Despite decades of mitigation efforts, emissions continue to rise, underscoring the urgency for scalable low-carbon energy solutions.

Microalgae have emerged as a promising biofuel feedstock due to their rapid growth, high photosynthetic efficiency, and ability to utilize wastewater while removing nutrients and organic pollutants^4^. Compared with terrestrial crops (1–6 t ha⁻¹ yr⁻¹), microalgae can achieve biomass productivities of 20–30 t ha⁻¹ yr⁻¹ under favorable conditions. Certain strains accumulate lipids up to 50–60% of their dry weight^5^, exceeding conventional oilseed crops such as soybean (~ 20%) and rapeseed (~ 40%). Wastewater-grown microalgae also reduce freshwater use and contribute to nutrient remediation, supporting circular-economy objectives^6^.

However, traditional lipid-extraction pathways are energy intensive, generate solvent waste, and leave non-lipid fractions underutilized. Thermochemical options such as pyrolysis or gasification require extensive drying, limiting their efficiency. Hydrothermal liquefaction (HTL) offers a more suitable alternative by directly converting wet biomass into bio-crude under subcritical water conditions, thereby avoiding energy-intensive drying. Yet, many existing HTL studies rely on laboratory-cultured algal strains, narrow reaction conditions, or limited fuel-quality characterization, leaving knowledge gaps regarding feedstock realism, process optimization, and integration with wastewater treatment systems^7–9^.

HTL converts biomass via hydrolysis and subsequent reactions decarboxylation, deamination, polymerization occurring at 250–374 °C and 10–25 MPa, producing bio-crude, an aqueous phase, gases, and solid char^10,11^. Process efficiency and product distribution are strongly influenced by reaction parameters such as temperature, residence time, pressure, catalysts, and the solvent environment^12–15^. Temperature (280–350 °C) critically governs depolymerization and deoxygenation; higher temperatures enhance conversion but increase secondary cracking and gas formation^16^. Residence times of 15–60 min can improve conversion but may also promote char formation at longer durations^17^. High pressure maintains water in the liquid phase, while catalysts (e.g., Na₂CO₃, metal oxides, zeolites) can enhance denitrogenation and deoxygenation and improve biofuel quality^18^.

Typical HTL of algal biomass yields 30–60 wt% bio-crude, along with an aqueous phase rich in soluble organics and nutrients, a CO₂-dominated gas phase, and solid char with potential valorization pathways. Most prior research has focused on single algal strains under simplified conditions, with limited emphasis on realistic mixed wastewater biomass or comprehensive fuel property assessment^19,20^.

This study addresses critical gaps in HTL research using wastewater-grown algal biomass. Its novelty lies in employing a realistic mixed algal-cyanobacterial feedstock naturally grown in municipal wastewater, which simultaneously couples nutrient removal with renewable fuel production. It systematically investigates the influence of key HTL parameters, including temperature, residence time, and algae-to-water ratio, on biofuel yield and quality, identifying conditions that maximize fuel potential. In addition, the study provides a comprehensive characterization of the resulting biofuel, including its chemical composition and fuel properties, demonstrating its suitability as a sustainable energy source. Collectively, these contributions offer a practical framework for integrating HTL technology into wastewater-based biorefineries, advancing both process efficiency and environmental sustainability compared to previous studies.

## Materials and methods

### Materials

#### Algal community structure

The high-rate algal pond (HRAP), constructed at the Zenin wastewater treatment plant in Giza, served as a system for both **municipal** wastewater treatment and microalgae cultivation. Aluminum sulfate (Al₂(SO₄)₃) was employed as a coagulant to facilitate the aggregation of microalgae prior to harvesting. Following coagulation, the biomass was subjected to solar drying under controlled conditions, with temperatures maintained between 40 and 45 °C. The microalgae biomass was preserved at a storage temperature of 0–4 °C to maintain its stability and prevent degradation^21^. The algal **Community structure** in HRAP dominated with the species composition presented in Table 1. Both qualitative and quantitative assessments of the algal community were carried out according to the standard key for freshwater algae identification (APHA2017 standard methods)^22^. Microscopic analysis was performed using an Olympus X3 microscope (Olympus Corporation, Tokyo, Japan), and algal enumeration was conducted with a Sedgwick-Rafter counting cell^23^. The proximate composition of the harvested algal biomass, summarized in Table 2, was used to characterize the feedstock specifications for subsequent hydrothermal liquefaction experiments^24^.

**Table 1:** Species composition of the algal consortium harvested from the HRAP.

Microalgal species	Relative abundance (%)
*Microcystis aeruginosa*	52.00
*Scenedesmus quadricauda*	20.00
*Pediastrum gracillimum*	17.00

**Table 2:** Proximate and ultimate analysis of municipal wastewater-grown mixed algal biomass.

Component	Analysis results (wt%)
Total carbohydrates	14.20
Total lipids	3.70
Total protein	11.00
Moisture content	6.61
Ash content	30.00
%Ca	25.14
%Al	5.15
%Cl	5.19
%Fe	7.52
%K	3.60
%P	2.52
%S	11.00
%Si	5.80
%Ti	0.75

#### Dichloromethane

Dichloromethane was purchased from Acros organics, Fisher scientific company, Germany. The DCM has a concentration of ≥ 99.8%, contains amylene as stabilizer, and suitable for HPLC grade. The molecular weight of 84.9 and the empirical formula is CH_2_Cl_2_.

### Methods

#### Hydrothermal liquefaction of microalgal biomass

**Fig. 1:**
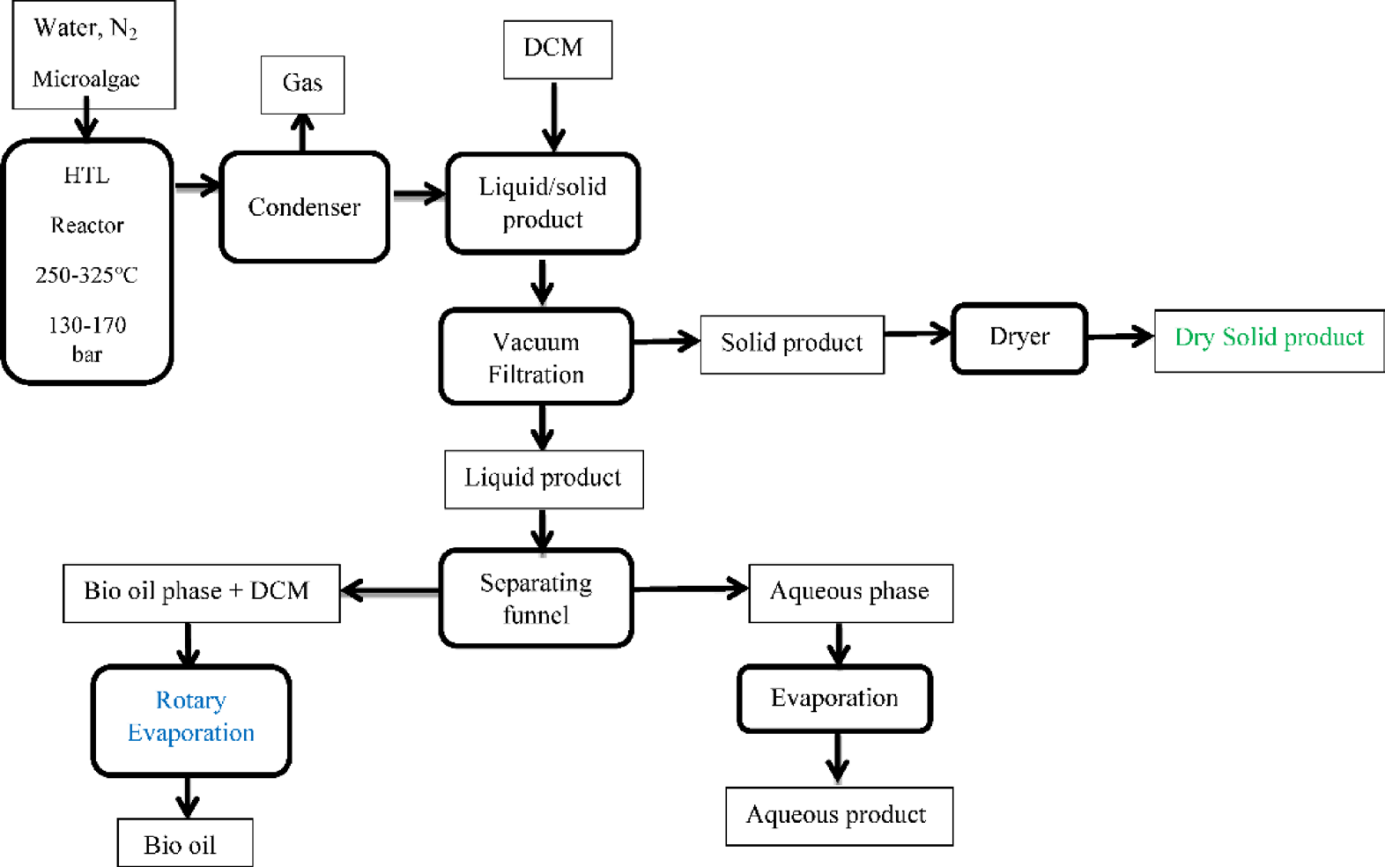
Streamlined process diagram of hydrothermal liquefaction.

According to Fig. 1, the hydrothermal liquefaction of microalgal biomass was executed in a 450 mL stainless steel high pressure and high temperature batch reactor manufactured by Parr instruments. The system was equipped with a pressure gauge and thermocouple, both connected to a control unit. After loading the feedstock into the reactor, it was sealed and purged with nitrogen gas to establish an inert environment. Heating was applied using an oven integrated with an oscillation mechanism to promote internal agitation. Table 3 illustrates that the experimental conditions for the hydrothermal liquefaction process were selected based on a combination of precedent literature and preliminary experimental trials to identify meaningful ranges for each of the three optimizing parameters: reaction temperature, residence time, and biomass-to-water ratio. Experimental temperatures ranged from 250 °C to 350 °C, with reaction times between 15 and 60 min, and corresponding reaction pressure. Biomass-to-distilled water ratios were varied from 1:5 to 1:20, all under nitrogen pressure from 80 to 280 bar^4,25^. The sequence of experiments was designed to vary one at a time while holding the other two constant, following a univariate optimization approach rather than a full factorial or multivariate design. This approach allowed us to isolate the individual effect of each parameter on biofuel yield and quality while maintaining experimental feasibility.

**Table 3:** Operating conditions of hydrothermal liquefaction process of microalgal biomass.

Test	Temperature, ℃	Time, min	Microalgae/water ratio, (w/w)	Pressure, bar
A1	325	30	(1/10)	280
A2	300	30	(1/10)	240
A3	275	30	(1/10)	180
A4	250	30	(1/10)	125
A5	250	60	(1/10)	120
A6	250	15	(1/10)	135
A7	250	45	(1/10)	120
A8	250	30	(1/5)	130
A9	225	30	(1/10)	80
A10	250	30	(1/15)	140
A11	250	30	(1/20)	115

Before each experiment, the reactor was flushed and pressurized with nitrogen gas (N₂) to ensure proper working pressure. Preliminary tests helped determine the necessary starting pressure for different test temperatures (ranging from 130 to 170 bar). As temperature increases, the system pressure naturally rises, so higher temperatures required lower initial pressures, though the relationship is not strictly linear. After each reaction, the system was cooled using an ice bath to bring it back to room temperature. Gaseous products were collected and measured first. Then, the reactor was opened, and the mixture was transferred into dichloromethane to help separate the biofuel from the aqueous and solid phases. The reaction products were divided into three phases: gas, liquid, and solid and the DCM formed a separate layer containing organic compounds (biofuel), while the aqueous and solid materials settled separately. Solid particles were filtered using a vacuum pump connected to a 0.45 μm, 47 mm cellulose nitrate membrane, then dried at 105 °C. The remaining two liquid layers were separated with a 2-liter separating funnel. Biofuel was extracted from the dichloromethane using a rotary evaporator at 60 °C, and the aqueous layer was evaporated at 100 °C to retrieve minerals and water-soluble compounds^17^.

#### Characterization of microalgal biomass and biofuel

**Elemental composition** (C, H, N, O, and S) of the HTL products was performed using a Vario EL III CHNS elemental analyzer (elementar, Germany). The system utilized helium as the carrier gas and oxygen as the oxidizing gas. Analyses were conducted at 1150 °C under a pressure of 1.20 Pa, employing a thermal conductivity detector (TCD). Two analytical procedures were required: one for the quantification of C, H, N, and S, and another for oxygen determination. The higher heating value (HHV) of the biofuel was obtained using the Dulong-Berthelot formula:

$$\:\mathrm{H}\mathrm{H}\mathrm{V}(\mathrm{D}\mathrm{u}\mathrm{l}\mathrm{o}\mathrm{n}\mathrm{g})\:=\:0.3383\mathrm{C}\:+\:1.443\mathrm{H}\:-\:0.1804\mathrm{O}\:+\:0.0942\mathrm{S}$$

The percentage of energy recovery (%ER) was estimated as follows^26,27^:

$$\:\mathrm{\%}\mathrm{E}\mathrm{R}\:=\frac{\mathrm{H}\mathrm{H}\mathrm{V}.\mathrm{b}\mathrm{i}\mathrm{o}-\mathrm{o}\mathrm{i}\mathrm{l}\:\times\:\:\mathrm{Y}\mathrm{i}\mathrm{e}\mathrm{l}\mathrm{d}.\mathrm{b}\mathrm{i}\mathrm{o}-\mathrm{o}\mathrm{i}\mathrm{l}\:\:}{\:\mathrm{H}\mathrm{H}\mathrm{V}.\mathrm{m}\mathrm{i}\mathrm{c}\mathrm{r}\mathrm{o}\mathrm{a}\mathrm{l}\mathrm{g}\mathrm{a}\mathrm{e}}$$

**Thermogravimetric analysis (TGA)** of the biofuel was conducted at the microanalytical center, faculty of science, Cairo university, Egypt, utilizing a SHIMADZU TGA-50 instrument (Japan). The analysis employed nitrogen as an inert carrier gas at a flow rate of 30 ml min^− 1^. A sample mass of 39.40 mg was used, with the temperature programmed to increase from 34 °C to 602 °C at a constant heating rate of 10 °C per minute^28^.

**Gas chromatography–mass spectrometry (GC/MS) analysis** was conducted with a thermo scientific trace GC ultra-equipment in conjunction with an ISQ single quadrupole mass spectrometer. A TG-5MS fused silica capillary column (0.251 mm internal diameter, 30 m in length, and 0.1 μm film thickness) was employed for the analysis. The system was operated under the following conditions: electron ionization at 70 eV, employing helium as the carrier gas at a steady flow rate of 1 ml min^−1^. A temperature of 280 °C was maintained for both the injector and the transfer line. After starting the oven at 45 °C (held for 2 min), increasing to 150 °C at 7 °C min^−1^, ramping up to 270 °C at 5 °C min^−1^ (held for 2 min), and then ramping to 310 °C at 3.5 °C min^−1^ with a final hold of 10 min, the temperature program concluded at 310 °C at 3.5 °C min^−1^. Compound identification was achieved by comparing retention times and mass spectra with those in the NIST WILLY library. The percentages of peak areas were used for quantification^29^.

## Results and discussion

### Effect of reaction time on product yields

**Fig. 2:**
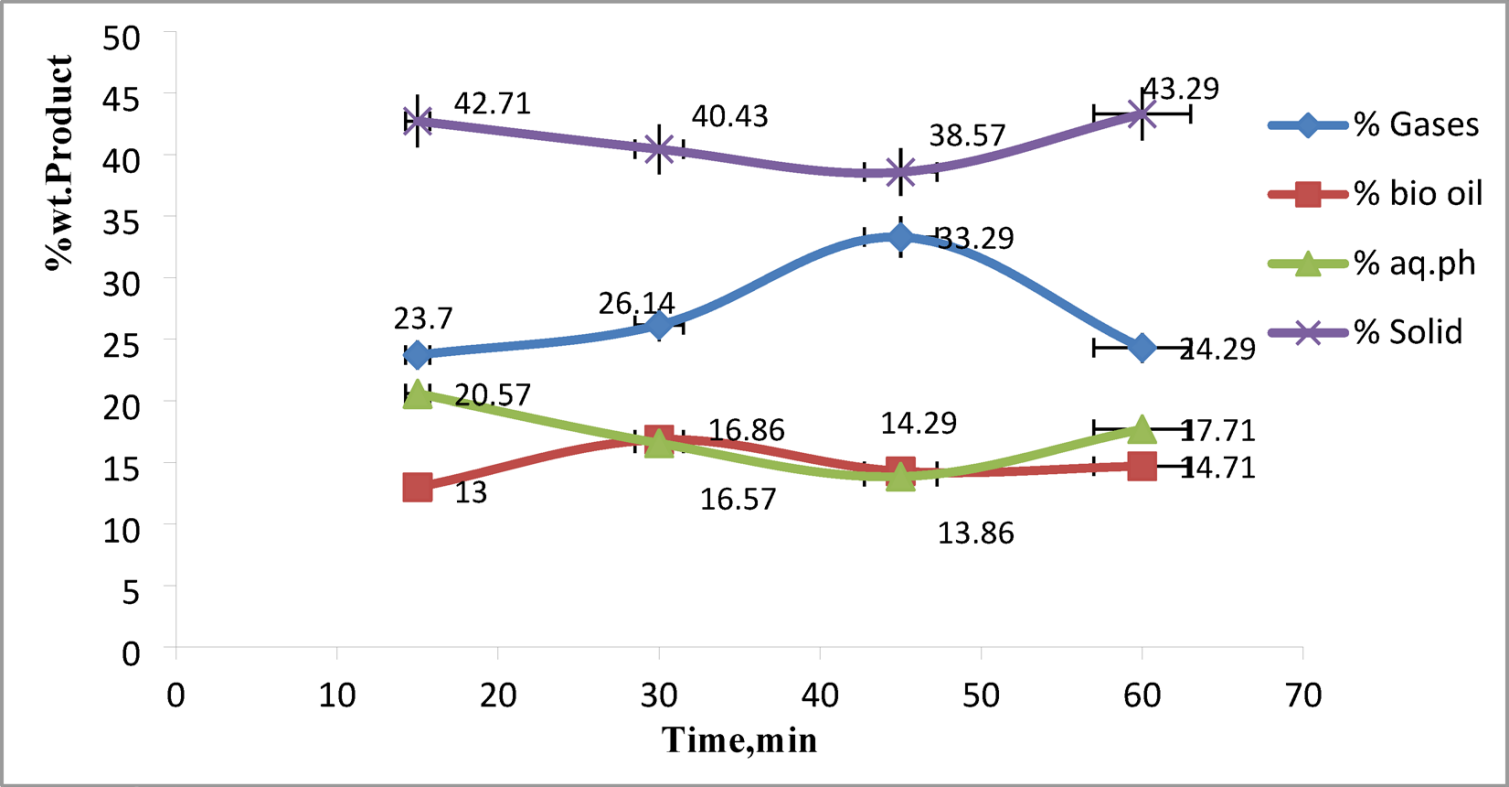
Biofuel yield of hydrothermal liquefaction process of microalgae at 250℃ and 1/10 algae-to-water at different time intervals.

As illustrated in Fig. 2, experiments were performed at a constant temperature of 250 °C and an algae-to-water ratio of 1:10, with varying reaction times. The biofuel yield increased from 13.00% to 16.86% by weight as the reaction time extended from 15 to 30 min. However, further extension of the reaction time beyond 30 min led to a decline in yield, dropping to 14.29%. This decrease is likely due to the breakdown of biofuel compounds into gaseous or aqueous products during prolonged exposure. Similar trends have been noted in previous studies, which identify 30–60 min as the ideal time frame for achieving maximum biofuel output^17,30^. In terms of gas formation, the yield increased from 23.70% at 15 min to a peak of 33.29% at 45 min, then declined to 24.29% at 60 min. Concurrently, yields of the solid and aqueous phases decreased from 42.71% to 20.50% to 38.50% and 13.86%, respectively, between 15 and 45 min. After 45 min, both solid and aqueous fractions began to increase again, reaching 43.29% and 17.71%, respectively.

Reaction time, therefore, plays a vital role in the efficient and cost-effective conversion of algal organics into biofuel. Notably, some researchers recommend 30 min as the optimal duration for maximizing biofuel production^31^. This may be attributed to secondary reactions, such as repolymerization and condensation of biofuel intermediates into heavier, more recalcitrant compounds that partition into the solid residue, and increased solubilization of oxygenated compounds into the aqueous phase at prolonged residence times. Similar trends have been reported in the literature. For example, Akhtar & Amin (2011) observed that extending reaction time beyond the optimal point led to char formation and higher solid yields due to polymerization of reactive intermediates^32^. Likewise, Jena et al. (2011) reported an increase in the aqueous phase yield at longer residence times, which they attributed to the further degradation of biofuel components into water-soluble products^33^. Therefore, our findings are consistent with previous studies and indicate that excessive reaction times promote undesirable secondary reactions that reduce biofuel yield while increasing solid and aqueous products.

The findings support the view that reaction times longer than 30 min are counterproductive for biofuel production, as they promote undesirable secondary reactions that shift the product distribution toward gaseous, aqueous, and solid phases. From a process optimization perspective, this suggests that a residence time of around 30 min strikes a balance between maximizing liquid fuel yield and minimizing by-products. It is believed that, this time frame is not only technically favorable but also economically viable, as longer durations would increase energy input while reducing valuable oil output. Therefore, 30 min can be considered a practical benchmark for scalable HTL processes, although fine-tuning may still be required depending on algal species and reactor configuration.

### Effect of reaction temperature on product yields

**Fig. 3:**
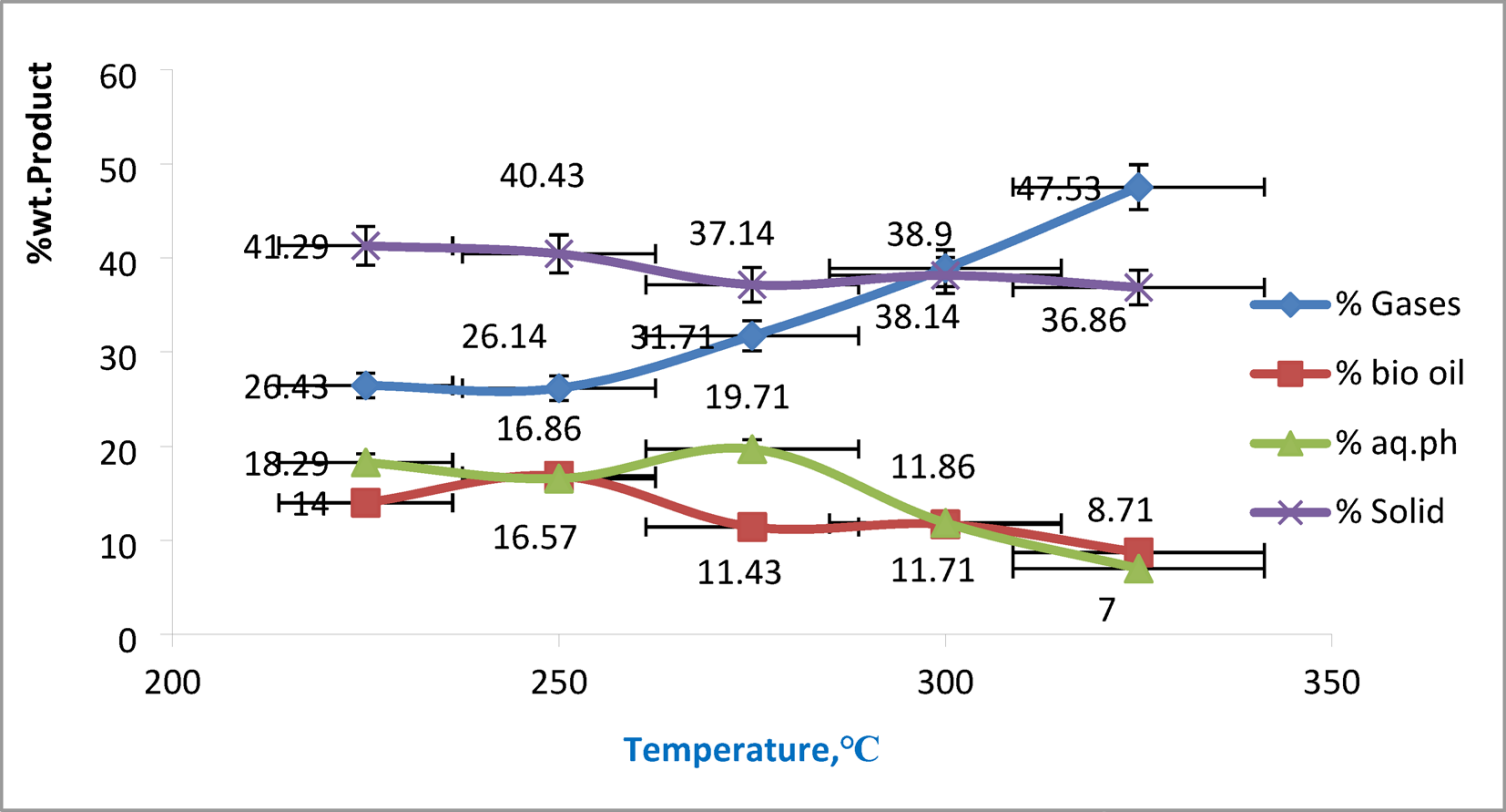
Biofuel yield of hydrothermal liquefaction process of microalgae at 30 min and 1/10 algae-to-water at different temperature ranges.

As shown in Fig. 3, experiments were conducted at varying temperatures (with a fixed reaction time of 30 min and algae-to-water ratio of 1:10). The biofuel yield increased from 14.00% to 16.86% by weight as the temperature rose from 225 °C to 250 °C. However, further temperature increases beyond 250 °C resulted in a yield reduction, with the biofuel output dropping to 11.71% and 8.71%. Simultaneously, solid and aqueous phase yields declined from 41.29% to 18.29% to 36.86% and 7.00%, respectively, as the temperature increased from 225 °C to 325 °C. This suggests that more intermediate water-soluble compounds were converted into biofuel and gaseous products. The gas yield notably rose from 26.14% to 31.71 and 47.53% over the same temperature range, reflecting greater organic matter conversion defined as the proportion of organics transformed into liquid and gaseous products relative to the original feedstock. Temperature plays a pivotal role in determining biofuel yield during hydrothermal liquefaction (HTL) of microalgal biomass. Selecting the appropriate temperature is essential for both safe operation in industrial applications. Numerous studies have examined how temperature influences both the quantity and quality of biofuel produced. Generally, optimal biofuel yields are achieved within the temperature range of 250–375 °C^16^. This is largely due to the fact that different chemical reactions are favored at various temperature levels. As temperature increases, water undergoes changes in its ionic properties, accelerating several critical reactions. Notably, near the critical point of water (374 °C and 22.10 MPa), the ionic product of water (Kw = [H⁺][OH⁻]) increases significantly, enhancing its ability to act as a catalyst in hydrolyzing complex organic compounds^8^. In this state, hot compressed water promotes hydrolysis reactions driven by H⁺ and OH⁻ ions, enabling the breakdown of proteins, polysaccharides, and lipids into smaller molecules via isomerization, depolymerization, and condensation. Beyond water’s critical point, elevated temperatures encourage gas formation through processes such as decarboxylation, cracking, steam reforming, and gasification of intermediate liquids or chars^34^.

At temperatures below 250 °C, macromolecules in microalgal biomass undergo partial hydrolysis and break down into smaller molecules. Between 250 °C and 300 °C, higher thermal energy allows these molecules to further disintegrate into monomers, enhancing biofuel production. At 325 °C, these small molecules begin to polymerize into oil, but exceeding this temperature can lead to secondary decomposition into gases and repolymerization into solids^17^. Research has identified 300–320 °C as an ideal temperature range for maximizing biofuel yield from microalgal biomass via HTL. While elevated temperatures can enhance biofuel production up to a point, excessively high conditions may negatively affect yield due to over-cracking and gas formation^8,35^.

The results indicate that temperatures around 250–300 °C offer the best balance between maximizing biofuel yield and limiting secondary degradation. While higher temperatures (> 300 °C) increase gas formation, they reduce liquid fuel recovery and shift the energy distribution toward less desirable fractions. From a process optimization perspective, this indicates that operating at moderate subcritical conditions is more economically viable and technically favorable for scalable HTL of algal biomass. It is believed that, the temperatures in the range of 250–300 °C should serve as a practical benchmark, although further optimization may depend on algal composition and reactor design.

### Effect of biomass to water ratio on product yields

**Fig. 4:**
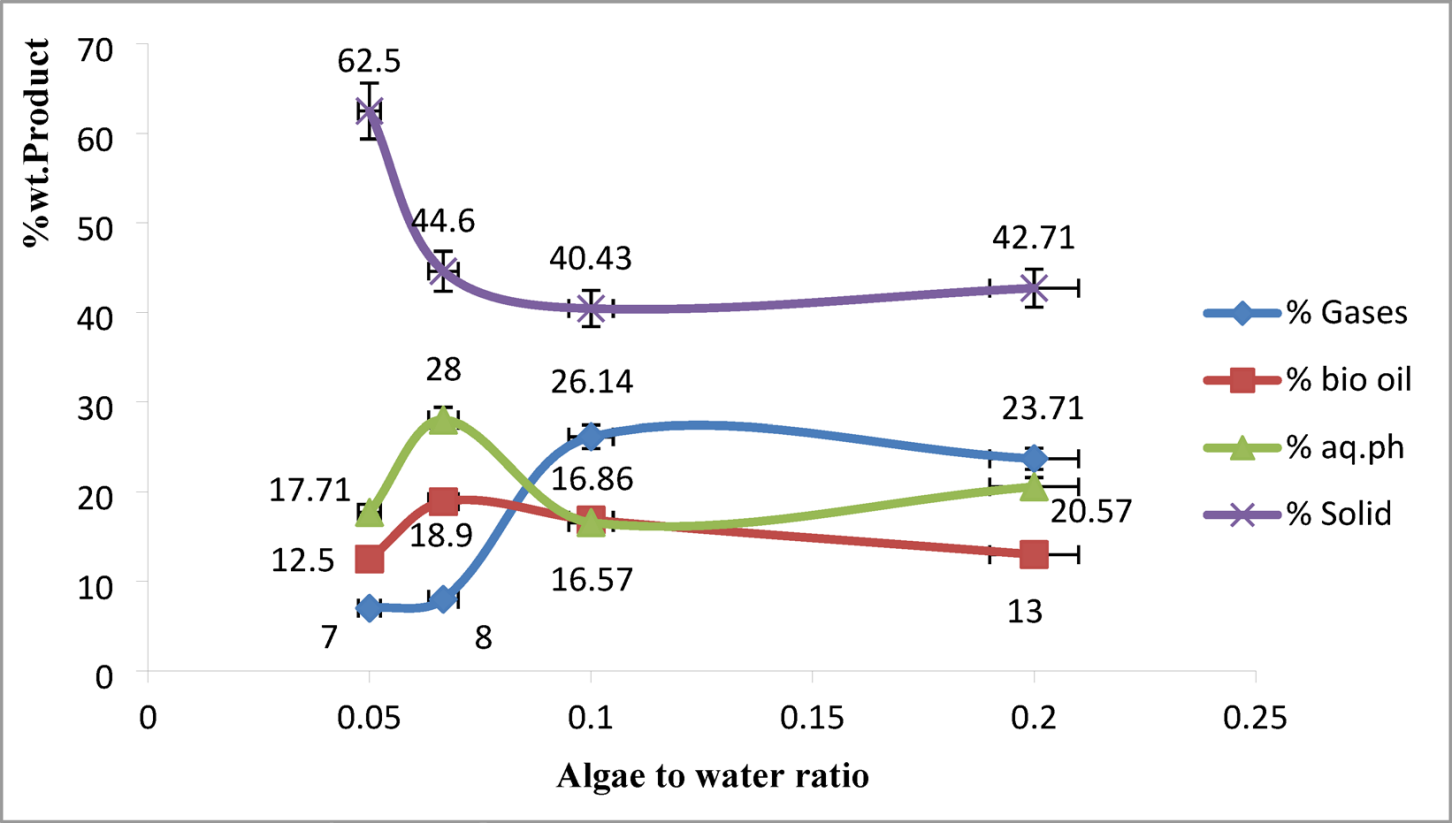
Biofuel yield of hydrothermal liquefaction process of microalgae at 30 min and 250℃ at different algae-to-water ratios.

The biomass-to-water ratio is a critical parameter in hydrothermal liquefaction (HTL) that directly affects product yield distribution, energy efficiency, and economic viability. In this study, as shown in Fig. 4, the biofuel yield increased from 12.50% to 16.57% as the ratio improved from 1:20 to 1:10, then declined to 13.00% at 1:5. While gas phase yield increased significantly from 7.00% to 26.14%, the solid fraction decreased from 62.50% to 42.71% with increasing biomass concentration. This reflects the shift in thermal degradation and conversion efficiency with varying slurry compositions.

As biomass concentration increases (e.g., from 1:20 to 1:5), more organic matter becomes available per unit of solvent, potentially enhancing product yields. However, both excessively high and low algal-to-water ratios can negatively impact biofuel recovery and overall process performance^16^. This is because higher biomass loading provides more lipids, proteins, and carbohydrates for thermochemical breakdown, leading to increased formation of bio-crude and gaseous products via hydrolysis, decarboxylation, and Maillard-type reactions^36^.

However, when biomass concentrations exceed the optimal range (e.g., beyond 1:10), reaction limitations arise. The thick slurry can lead to non-uniform heating, diffusion limitations, and reduced water availability for hydrolysis. These constraints hinder the breakdown of macromolecules and favor repolymerization and carbonization, increasing solid residue (char) formation^37^. Furthermore, insufficient water per unit of biomass may limit the formation of supercritical water species (e.g., hydronium ions), which are critical for cleaving peptide and glycosidic bonds, affecting the selectivity and extent of conversion^38^.

Mechanistically, moderate algal loadings (such as 1:10–1:15) facilitate effective hydrolysis of lipids, proteins, and carbohydrates, enabling their transformation into free fatty acids, gases (e.g., CO₂, NH₃), and biofuel precursors through reactions such as deamination, decarboxylation, and ketonization. However, at higher concentrations (e.g., 1:5), mass transfer resistance, insufficient water availability, and poor mixing limit the extent of liquefaction and favor repolymerization and char formation. On the other hand, overly diluted systems (e.g., 1:20) increase water demand and heating costs, leading to reduced energy efficiency and increased **municipal** wastewater volumes without proportional improvements in yield^18^.

The key limitation at low biomass loading (e.g., 1:20 or higher water content) is the dilution of energy density, leading to higher energy consumption per unit of biofuel produced. On the other hand, at high loadings (e.g., 1:5), reactor clogging, stirring inefficiency, and incomplete liquefaction may occur. The findings in this study confirm that 1:10 represents a technically and economically optimal ratio, ensuring sufficient reactant availability, uniform hydrothermal conditions, and balanced energy input-output efficiency. Future work should investigate continuous flow reactors and co-solvent systems to overcome the mass transfer constraints observed at higher loadings. It is to be noticed that used pressure is not regulate, but is adjusted according to the reaction condition.

### Characterization of microalgal biomass and biofuel product

**Fig. 5:**
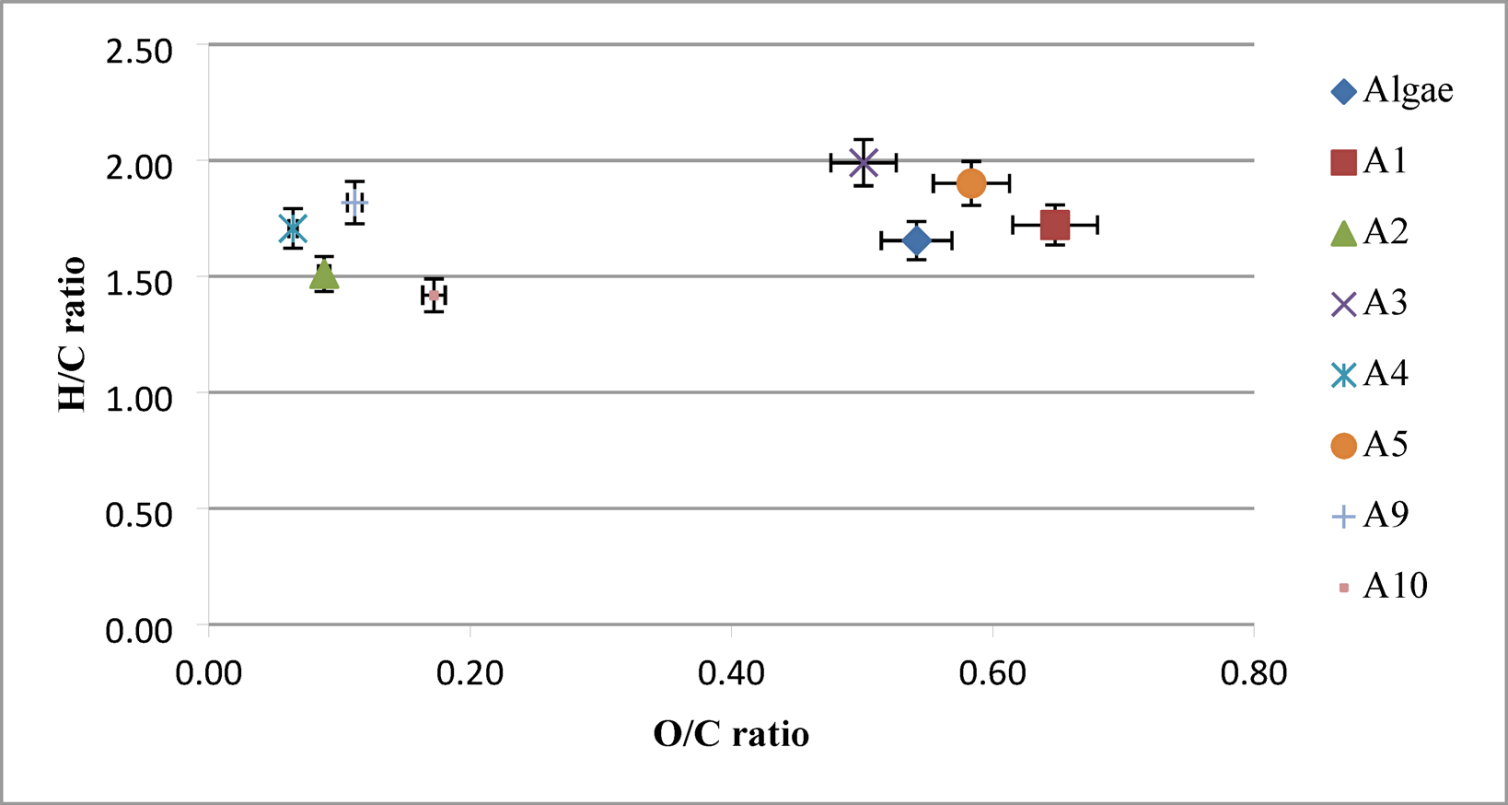
Van Krevelen’s plot of the feedstock and bio-oil from HTL.

**Elemental analysis** is a key characteristic of Biofuels, directly influencing their energy content and fuel quality^35^. According to Table 4, which compares the elemental compositions, higher heating values (HHVs), and energy recovery (ER) of microalgae and the corresponding Biofuels produced through hydrothermal liquefaction (HTL) under various operating conditions. According to Fig. 5, the elemental data were further analyzed using a Van Krevelen diagram. Among the experimental conditions studied, A4, A9, and A2 produced Biofuels with the most favorable elemental ratios specifically, high H/C ratios of 1.70, 1.82, and 1.51, and low O/C ratios of 0.06, 0.11, and 0.09, respectively indicating superior quality. The HHVs of the Biofuels were significantly improved compared to the raw algal biomass, approaching values typical of petroleum-based fuels (~ 43 MJ·kg⁻¹)^17^. The highest HHVs were observed under conditions A4 (42.93 MJ·kg⁻¹) and A9 (40.59 MJ·kg⁻¹), which also corresponded to the highest H/C ratios. High carbon (C) and hydrogen (H) contents indicate a higher energy density and better combustion characteristics, essential for transportation fuels. The observed C and H values correspond with a higher heating value (HHV) of 42.93 MJ.kg⁻¹ in A4, comparable to conventional fossil fuels. However, the presence of nitrogen may affect combustion emissions and fuel stability, suggesting the need for further upgrading. Low oxygen (O) content is desirable in A4 (6.98%), as oxygenates in biofuel reduce thermal stability and energy content. A lower oxygen percentage suggests less need for extensive upgrading or hydrodeoxygenation during refining^17^.

**Table 4:** Elemental analysis of microalgal biomass and biofuel product.

Sample	Analysis results (wt%)								HHV (MJ·kg⁻¹)	% Energy recovery
	C	H	*N*	S	O	*N*/C	O/C	H/C		
Microalgal biomass	49.32	6.80	6.64	1.61	35.63	0.12	0.54	1.65	20.22	-
A1	45.31	6.50	8.12	0.95	39.13	0.15	0.65	1.72	17.74	7.64
A2	80.12	10.09	0.32	0.04	9.43	0.00	0.09	1.51	39.97	23.14
A3	48.24	8.00	10.17	1.36	32.24	0.18	0.50	1.99	22.18	12.59
A4	81.30	11.56	0.15	0.01	6.98	0.00	0.06	1.71	42.93	35.79
A5	46.46	7.36	8.88	1.14	36.16	0.16	0.58	1.90	19.92	14.49
A9	76.57	11.60	0.41	0.01	11.41	0.00	0.11	1.82	40.59	28.10
A10	69.78	8.25	1.63	4.31	16.03	0.02	0.17	1.42	33.03	30.86

Conversely, the lowest HHVs were recorded for experiments A1 and A5. Energy recovery followed a similar trend, being strongly influenced by both the H/C and O/C ratios. The highest ER was observed in condition A4, while the lowest was in A1. Notably, increasing the reaction time from 30 to 60 min had a detrimental effect on biofuel quality. For example, in experiments A9 and A5, the H content decreased from 76.57% to 46.46%, while the O content increased from 11.41% to 36.16%, resulting in declines in both HHV (from 40.59 to 19.92 MJ·kg⁻¹) and ER (from 28.1% to 14.49%). Similarly, increasing the algae-to-water ratio from 1:10 to 1:15 adversely affected biofuel quality. Under experimental conditions A9 and A10, the H content decreased from 76.57% to 69.70%, HHV dropped from 40.59 to 33.00 MJ·kg⁻¹, and O content rose from 11.41% to 16.00%. Overall, elevated oxygen content, whether due to extended reaction time or higher algae-to-water ratios, corresponded to reduced carbon and hydrogen content in the Biofuel. Based on the findings of this study, the optimal reaction temperature was determined to be 250 °C. The elemental analysis shows that wastewater-grown algal biomass initially contains low carbon and high oxygen and nitrogen due to its protein-rich mixed algal–cyanobacterial composition. After HTL, the biofuel exhibits a marked increase in carbon content (up to ~ 75 wt%) and a major decrease in oxygen (5–10 wt%), confirming effective deoxygenation through decarboxylation and dehydration pathways. These results closely match reported ranges in the literature, where microalgal biocrudes typically contain 70–78 wt% C and 8–12 wt% O. The resulting H/C ratios (1.5–1.8) also fall within expected values for partially upgraded HTL biocrude, reflecting the formation of more hydrogen-rich hydrocarbon structures relative to the raw feedstock^39^. These compositional characteristics also influence the viscosity and flow behavior of the Biofuel, which are critical for pumping, atomization, and downstream upgrading processes such as hydrodeoxygenation. Therefore, understanding these parameters is essential not only for evaluating fuel quality but also for designing effective refining strategies to produce drop-in fuels suitable for conventional engines.

**Thermogravimetric analysis TGA** was employed to assess the boiling point distributions of microalgal biomass and Biofuels produced under various HTL conditions. TGA serves as a small-scale distillation technique; while some thermal degradation may occur, it provides a useful approximation of the boiling range of heavy oils^35^. In this analysis, samples were heated under a nitrogen atmosphere from 30 °C to 600 °C and Table 5 illustrates the boiling point distributions derived from TGA data. Crude biofuel exhibited a substantial fraction of high-boiling-point compounds, with over 30% of the material boiling above 350 °C. In contrast, Biofuels generated via HTL contained a higher proportion of lower-boiling components (i.e., below 350 °C) compared to the raw microalgae feedstock. This shift is attributed to thermal cracking and molecular breakdown during HTL, which enhances the formation of more volatile compounds. Literature indicates that Biofuels derived from organic biomass typically contain 38.40–53.10% of components with boiling points between 250 and 350 °C comparable to diesel fuel and 12.00–21.70% of fractions below 250 °C, similar to gasoline^40^. Furthermore, HTL-derived Biofuels generally comprise approximately 50% of components with boiling points exceeding 300 °C, with the most significant mass loss occurring around 300–350 °C^41^. Table 5 categorizes the Biofuels into five distillation cuts, analogous to gasoline, kerosene, diesel, motor oil fractions, and minor products over 600 °C. The highest yields of low-boiling fractions were obtained under conditions A1 (61.00%) and A2 (56.00%), which involved elevated temperatures of 325 °C and 300 °C and shorter residence times of 30 min. Conversely, the highest yields of high-boiling fractions were observed in experiments A10 (51.50%) and A5 (51.00%), conducted reaction conditions of temperatures (250 °C) and longer reaction times (60 min). Overall, HTL proves to be an efficient thermochemical conversion method for producing high-quality liquid biofuels from algal biomass. By optimizing operating conditions, the process enhances fuel properties and boiling range distributions, contributing to sustainable energy development and reduced environmental impact.

**Table 5:** Boiling point range distribution of microalgal biomass and biofuel under different conditions.

Sample	Mass fraction (wt%)				
	Bio-gasoline 30–170 ℃	Bio-kerosene 170–270 ℃	Bio-diesel 270–350 ℃	Bio-Motor oil 350–600 ℃	> 600 ℃
Microalgal biomass	8.06	8.41	17.21	31.37	34.96
A1	11.28	37.93	11.87	38.86	0.10
A2	10.60	34.69	10.38	40.32	3.99
A3	7.98	31.81	13.12	46.60	00.48
A4	11.37	29.41	9.71	47.46	2.02
A5	7.17	26.83	14.68	51.05	00.25
A9	12.10	29.92	8.96	49.08	00.00
A10	10.92	30.13	6.80	51.54	00.00

The TGA results in this study show a clear shift toward lower-boiling fractions in HTL-derived Biofuels compared to the raw algal biomass, consistent with published HTL studies. Previous research reports 38–53% distillates in the 250–350 °C diesel range and 12–22% in the gasoline range, while our optimized conditions produced up to 61% low-boiling fractions, indicating comparatively higher conversion efficiency. This improvement may be linked to the biochemical richness of wastewater-grown mixed algae and the shorter residence times employed. However, because TGA provides only mass-loss data and may involve overlapping degradation events, the accuracy of boiling-range estimation remains limited^40^. Thus, while our findings align with literature trends, they underscore the need for complementary methods such as simulated distillation or GC–MS.

While TGA is an effective method for determining the thermal stability and breakdown behavior of biofuel and solid wastes, it has significant limitations when used with complex biomass-derived materials. One major disadvantage is that TGA simply offers mass loss data and does not identify the precise chemical components formed during decomposition. As a result, it is unable to identify overlapping degradation processes caused by distinct components such as lipids, proteins, and carbohydrates. Furthermore, TGA typically operates in a controlled inert or oxidative environment, which may not accurately simulate real-world combustion or processing conditions. Volatile components with comparable thermal behavior may co-evaporate, resulting in underestimating or misinterpretation of specific fractions. Finally, the limited sample size and consistent heating rate may not effectively reflect large-scale thermal processes, constraining the generalization of laboratory TGA findings to industrial contexts.

**Fig. 6:**
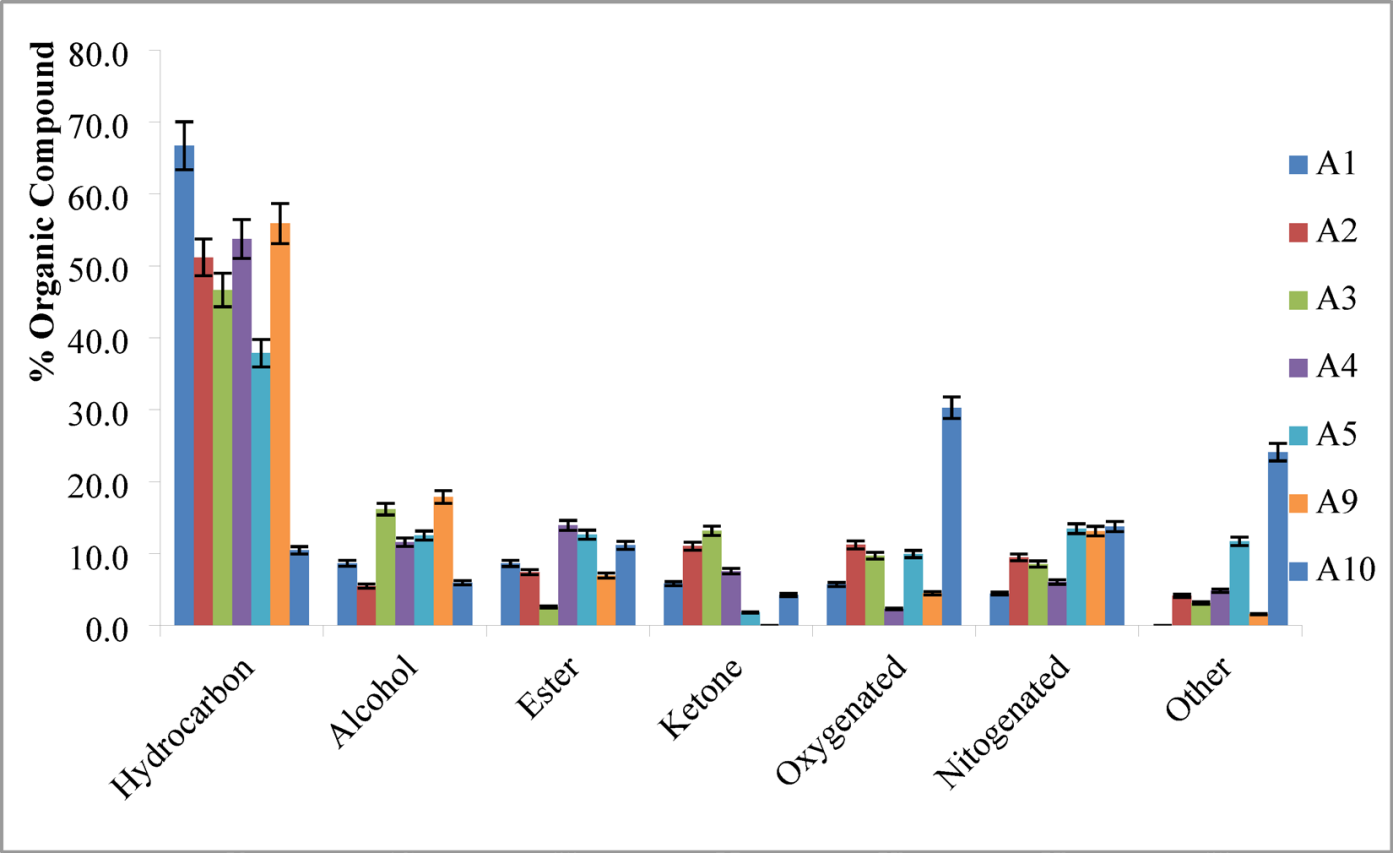
Different groups of chemical compounds in the bio-oil detected by GC-MS at different reaction conditions.

**Gas chromatography–mass spectrometry (GC–MS)** was used for identification purposes of the primary volatile compounds present in Biofuels produced via HTL of algal biomass under varying operating conditions. These conditions included reaction temperatures ranging from 225 to 325 °C, algae-to-water ratios from 1:5 to 1:20, and residence times between 15 and 60 min. The GC inlet temperature was maintained at 300 °C, which allowed approximately 80% of the biofuel to volatilize. Thus, the results represent a semi-quantitative analysis of the volatile fraction. Figure 6 illustrates the compounds in the Biofuels which were classified based on their functional groups into hydrocarbons, organic acids, nitrogen-containing compounds, phenols, esters, and oxygenated compounds (e.g., aldehydes, alcohols, ethers, and ketones). GC–MS data were used to compute peak area percentages, enabling a comparative analysis of chemical group distributions across different experiments. The nitrogen compounds included pyrroles, indole derivatives, and pyridines; oxygenates consisted of aldehydes, alcohols, and organic acids; while hydrocarbons ranged from aliphatic long-chain compounds (C6–C40) to cyclic structures such as cyclopentadecane. Aliphatic hydrocarbons are desirable for biofuel applications due to their high energy content and compatibility with conventional fuels. The highest yields of aliphatic hydrocarbons were observed in experiments A1 (66.70%), A9 (55.90%), A4 (53.70%), A2 (51.20%), and A3 (46.60%). In contrast, experiments A10 and A5 yielded the lowest percentages of 10.50% and 37.80%, respectively. Among process variables, temperature was found to be the most significant. Reaction temperatures above 275 °C, such as in experiments A1–A3, led to a decrease in aliphatic hydrocarbon content, whereas temperatures below 275 °C, as seen in A4 and A9, resulted in higher yields. Variations in reaction time and algae-to-water ratio contributed to differences in the observed results. Extending the reaction time from 30 to 60 min (A9 to A5) reduced the aliphatic hydrocarbon content from 55.90% to 37.80%. Similarly, increasing the biomass-to-water ratio from 1:15 to 1:10 (A9 to A10) significantly reduced the aliphatic hydrocarbon yield. These results indicate that higher temperatures, shorter residence times, and lower biomass loading enhance hydrocarbon production and improve overall biofuel quality. HTL also promotes the formation of oxygenated compounds via oxidation and condensation reactions. While these oxygenates generally impair combustion performance and engine efficiency, they are valuable in other industries. For example, isophytol a fragrance compound detected in experiment A10 is widely used in cosmetics, soaps, and detergents^29,36^. Hence, experiment A10 is considered optimal for producing value-added chemicals with 30.30% oxygen content, while experiments A9 and A4 are more favorable for high-quality biofuel production with oxygenated compounds 4.50% and 2.30%.

Nitrogen content in Biofuels, derived from protein decomposition during HTL, adversely affects fuel quality and stability. Favorable conditions for reducing nitrogen compounds include lower temperatures, shorter reaction times, and higher algae-to-water ratios. For instance, nitrogen content decreased from 9.50% to 6.10% when reaction temperature was reduced according to experiments A2 to A4. Similarly, nitrogen content decreased from 13.80% to 13.10% with increasing the algae-to-water ratio from A10 to A9 and reducing reaction time from A5 to A9 also lowered nitrogen compound concentrations from 13.50% to 13.10%. Esters, alcohols, and organic acids were also identified, with their presence varying across reaction conditions. Compounds such as 9,12,15-octadecatrienoic acid esters, 1-heptadecanol, and piperidinecarboxylic acid were detected. The formation of ketones e.g., crotonophenone and pentadecanone resulted from ring-opening and cellulose degradation, but their presence is generally undesirable due to their association with decarboxylation reactions and reduced fuel stability. Achieving an optimal distribution requires balancing the operating parameters of hydrothermal liquefaction such as temperature, residence time, and algae-to-water ratio to favor the production of desirable hydrocarbons while minimizing undesired oxygenated, nitrogenous, or overly heavy compounds. Excessively severe conditions can lead to repolymerization and char formation, reducing biofuel quality, whereas too mild conditions result in incomplete conversion. Therefore, careful optimization is essential to maximize hydrocarbon yield in the desired range while maintaining fuel quality and minimizing emissions potential.

Carbon chain length is a critical parameter in assessing fuel performance^42^. Shorter chains (C8–C16) are typical of gasoline and jet fuels, offering high volatility and faster combustion, while longer chains (C16–C22) resemble diesel, providing higher energy density but potentially increased emissions^43^.

**Fig. 7:**
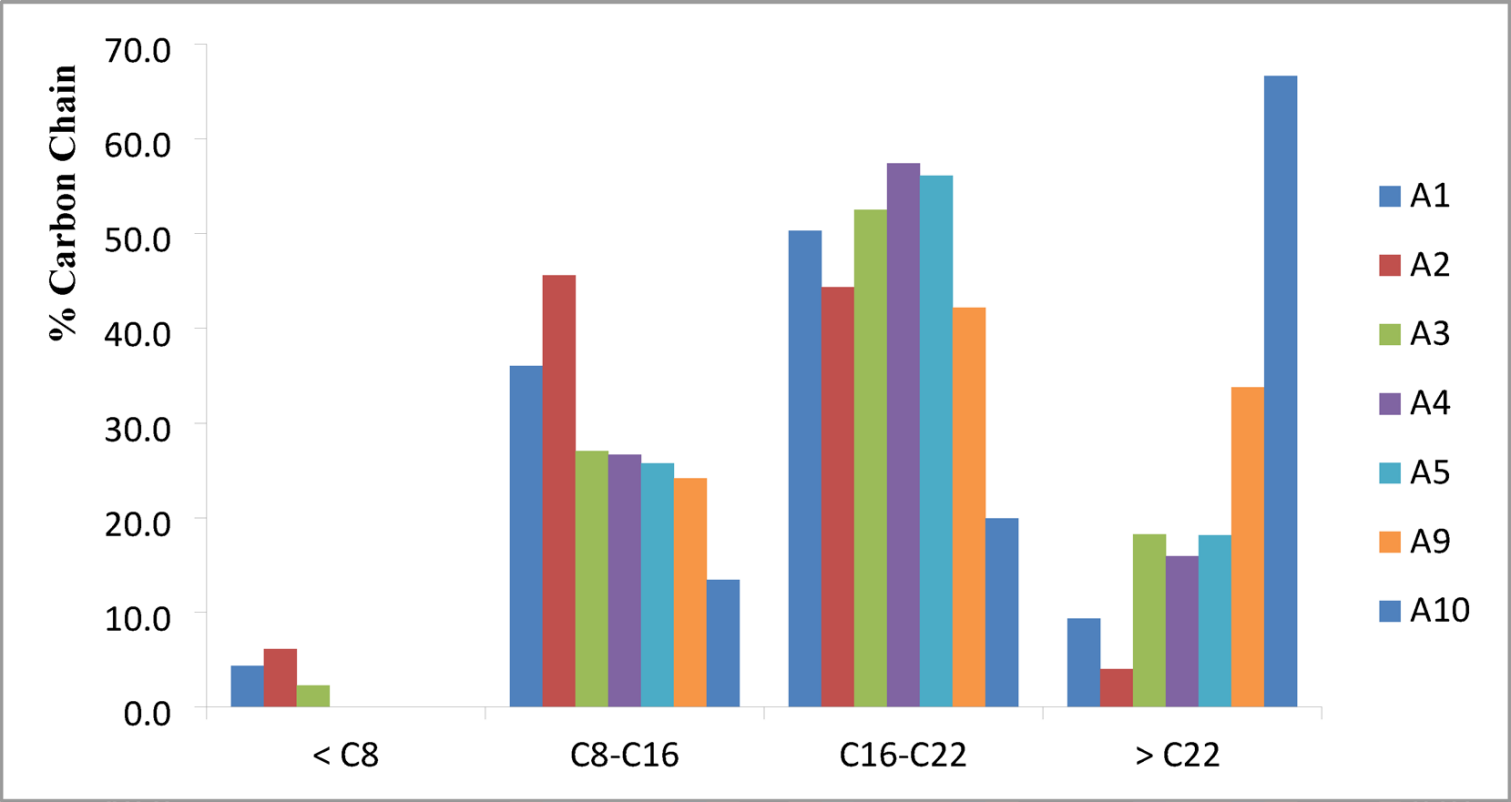
Carbon chain ranges of chemical compounds in the bio-oil detected by GC-MS.

Figure 7 illustrates and categorizes the compounds into distillation cuts: gasoline (> C8) , jet fuel (C8–C16), diesel (C16–C22), and motor oil (> C22). Preferred biofuel compositions fall within the C8–C22 range, corresponding to jet and diesel cuts. Experiments A2 and A4 produced the highest proportions of jet and diesel fractions, indicating superior fuel quality. In contrast, A10 exhibited the highest proportion (66.60%) of motor oil-range compounds (> C22), indicating less favorable fuel characteristics due to extended reaction times, reduced algae-to-water ratios, and lower temperatures. Thus, optimizing HTL conditions specifically by increasing reaction temperature, reducing residence time, and lowering biomass loading can enhance cracking processes; reduce oxygen and nitrogen content, and yield Biofuels with favorable fuel-like properties.

## Conclusions

This study demonstrates the feasibility of hydrothermal liquefaction (HTL) as an effective route for converting municipal wastewater-grown mixed algal–cyanobacterial biomass into Biofuel, integrating municipal wastewater treatment with energy recovery. Eleven HTL experiments were conducted across varying temperatures, residence times, and algae-to-water ratios to identify conditions that maximize yield and product quality. Optimal performance was achieved at 250–300 °C, 30 min, and a 1:10 ratio, producing biofuel rich in low-boiling aliphatic hydrocarbons suitable for fuel applications. The co-produced aqueous and solid phases were also found to contain recoverable nutrients, supporting their potential reuse within circular bioeconomy systems.The optimized biofuel exhibited an O/C ratio of 0.06, an H/C ratio of 1.71, an HHV of 42.93 MJ kg⁻¹, and an energy recovery of 35.79%. Product yields reached 16.86% Biofuel, 26.14% gas, and 40.43% solids. Despite these promising outcomes, challenges remain, including feedstock variability, secondary reactions at longer residence times, and limitations associated with small-scale operation. Future work should focus on scaling the process, evaluating economic and environmental performance, and enhancing efficiency through catalytic upgrading and energy integration. Advancing these areas will support broader implementation of HTL for sustainable resource recovery and renewable fuel production.

## Data Availability

The datasets used and/or analysed during the current study available from the corresponding author on reasonable request.

## Abbreviations

ER: Energy recovery
GC–MS: Gas chromatography–mass spectrometry
TGA: Thermogravimetric analysis
DCM: Dichloromethane
H/C: Hydrogen/Carbon
HHV: High Heating value
HTL: Hydrothermal liquefaction
Kw: Auto-ionization constant
